# Wild or Introduced? Investigating the Genetic Landscape of Cacao Populations in South America

**DOI:** 10.1002/ece3.71746

**Published:** 2025-07-22

**Authors:** Matheus Colli‐Silva, James Edward Richardson, José Rubens Pirani, Antonio Figueira

**Affiliations:** ^1^ Departamento de Botânica, Centro de Biociências Universidade Federal de Pernambuco Recife Brazil; ^2^ Royal Botanic Gardens, Kew Richmond UK; ^3^ School of Biological, Earth and Environmental Sciences University College Cork Cork Ireland; ^4^ Environmental Research Institute University College Cork Cork Ireland; ^5^ Faculty of Natural Sciences Rosario University Bogotá Colombia; ^6^ Departamento de Botânica Instituto de Biociências, Universidade de São Paulo São Paulo Brazil; ^7^ Centro de Energia Nuclear Na Agricultura Universidade de São Paulo Piracicaba Brazil

**Keywords:** cocoa, crop genomics, domestication, genetic resources, historical geography, RAD‐seq, Theobroma

## Abstract

Cacao ( *Theobroma cacao* ), the primary source for chocolate manufacturing, is native to the Upper Amazon basin. It was introduced into Mesoamerica by pre‐Columbian societies and later spread globally following European colonization, becoming a commercially significant crop. Today, cacao populations exist along a continuum from wild to naturalized and cultivated forms across the Tropical Americas, complicating efforts to distinguish genuinely wild populations from those influenced by human activity. Here, we investigate genomic diversity, population structure, and domestication signals in three groups using RAD‐sequencing: Upper Amazonian populations (including Contamana, Marañón, Iquitos and Nanay), the Guiana population, and the Amelonado variety introduced into Eastern Brazil in the 18th century. The Upper Amazonian populations exhibited the highest genetic diversity and limited evidence of recent selection, reaffirming their role as the primary genetic source of cacao. The Amelonado group displayed signatures of artificial selection, including reduced genetic diversity and evidence of balancing selection, consistent with its introduction to Bahia before its later expansion to West Africa. The Guiana population showed intermediate genetic diversity and tight clustering but minimal differentiation from Upper Amazonian populations, suggesting they could represent an isolated wild lineage rather than an introduced group. These findings highlight the complexity of cacao's domestication history, shaped by multiple independent selection events and long‐term human influence. Understanding this continuum is important for unraveling the species' evolutionary history for supporting conservation and breeding strategies for cacao, a crop of major economic and cultural importance.

## Graphical abstract

Cacao populations span a continuum from wild to domesticated forms, shaped by complex patterns of human influence and selection. Using RAD‐sequencing, we show that native Upper Amazonian populations harbor the highest genetic diversity, while the Guiana group likely represents an isolated wild lineage and Amelonado shows clear signals of artificial selection. These findings clarify cacao's evolutionary history and support efforts in conservation and breeding.

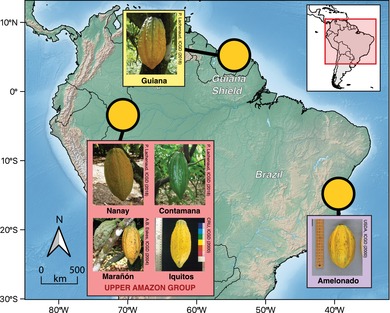

## 1 Introduction

Cacao ( *Theobroma cacao* L., Malvaceae) is a culturally and economically significant tree crop. While cacao serves as a fundamental ingredient in various products, its primary use is in chocolate manufacturing, a market that exceeded US$100 billion in retail value in 2021 (Bermudez et al. 2022). Revered as a sacred beverage in ancient Mesoamerica, cacao has shaped trade, culture, and human history for millennia, inspiring Linnaeus to name it as the “food of the Gods” (Schultes 1984). Today, cacao cultivation supports millions of smallholder farmers, particularly in West Africa and the Tropical Americas (Bermudez et al. 2022), and suffers increasing pressure from climate and land use change. The conservation and strategic use of cacao's genetic diversity are thus urgent priorities for crop improvement and resilience (Nieves‐Orduña et al. 2023).

The domestication history of cacao is intertwined with its long evolutionary trajectory. According to Richardson et al. (2015), *T. cacao* diverged from its most recent common ancestor over ten million years ago, undergoing dispersion influenced by natural events and human activity. Environmental shifts, such as those provided by the uplift of the Andes and Pleistocene climatic fluctuations, drastically altered the Amazon basin's ecosystems (Hoorn et al. 2010) and it may have affected natural selection processes. But human intervention also played a role in cacao's distribution and trait selection. For instance, Barrau (1979) and Schultes (1984) described that original people from Amazonia might have consumed the seed pulp, discarding the seeds—a practice likely driving artificial selection. Archaeological and genomic evidence suggests that the domestication of cacao began at least 5300 years ago in the Upper Amazon, where human populations likely began managing and selecting for favorable traits (Lanaud et al. 2024; Zarrillo et al. 2018). Over time, the seeds gained value for beverages and later for chocolate production in the late 19th century, prompting further selection for other favorable traits.

Cacao exhibits a significant morphological diversity, with pods and seeds varying in shape, size, color, weight, and chemical composition (Bucheli et al. 2001). Traditionally, these variations formed the basis to categorize cacao into two main groups, “Criollo” and “Forastero,” with a third hybrid group, “Trinitario” (Cheesman 1944). Although other classification systems have been proposed (e.g., Cuatrecasas 1964; Figueira et al. 1994; Pittier 1924; Preuss 1901), the Criollo‐Forastero system remains widely used, especially in agricultural contexts. But further studies have revealed that this classification would not fully encompass the genetic diversity present in the now eleven identified clusters (populations): Amelonado, Caquetá, Contamana, Criollo, Curaray, Guiana, Iquitos, Marañón, Nacional, Nanay, and Purús (see Motamayor et al. 2008; Osorio‐Guarín et al. 2017; Fouet et al. 2022). Additionally, Cornejo et al. (2018) demonstrated that the Criollo group was introduced into Mesoamerica approximately 3600 years ago from present‐day Ecuador. This classification has since been revised by genomic studies, which have refined the understanding of cacao's population structure and identified key clusters relevant for breeding and conservation (Gutiérrez et al. 2021; Nieves‐Orduña et al. 2023).

Among these groups, the Upper Amazon basin is recognized as the primary center of cacao's genetic diversity (Cheesman 1944; Cornejo et al. 2018; Fouet et al. 2022). While wild populations from this region often exhibit high nucleotide diversity, they also show variable inbreeding, with some individuals reaching homozygosity levels above 90% (Lopes et al. 2022; Silva et al. 2011). This variation raises questions about the distinctiveness and origins of other populations from Northern South America, particularly the Guiana group, which may represent either wild populations or human introductions (Colli‐Silva et al. 2024; Lachenaud et al. 2004; Lachenaud and Zhang 2008). Recent work has also shown that cacao's evolutionary dynamics extend beyond SNP‐level variation. For instance, Hämälä et al. (2020) demonstrated that polygenic adaptation can affect gene coexpression networks related to ecological traits such as flowering and water transport. Similarly, Hämälä et al. (2021) highlighted the role of structural variants–often deleterious but sometimes adaptive–in shaping population structure and gene expression, particularly through recombination suppression in low‐diversity regions.

The Amelonado variety was introduced into Bahia in the 18th century from Pará (Santos et al. 2015; Vello and Garcia 1971) and later spread to West Africa. The Amelonado group displays low genetic diversity and elevated inbreeding—traits linked to self‐compatibility and its narrow genetic base (Bartley 2005; Cope 1976; Santos et al. 2015). Although largely homogeneous, variation across Amelonado accessions suggests the influence of early introduction events and subsequent cultivation practices, such as farmer selection or propagation methods (Lopes et al. 2022).

In this study, we use RAD‐sequencing to examine genetic diversity, population structure, and domestication signals across three cacao groups: Upper Amazonian wild populations, the Guiana group, and the Amelonado variety introduced into Eastern Brazil. The Upper Amazonian group comprises four wild populations (Contamana, Marañón, Iquitos, and Nanay), which are treated as a single group here due to their shared wild origin and geographic proximity in the Upper Amazon region, consistent with established clustering in earlier studies (Cornejo et al. 2018; Fouet et al. 2022; Motamayor et al. 2008). By comparing these groups, we aim to clarify the boundaries between wild and introduced cacaos and assess whether Guiana accessions retain signatures of wild ancestry or reflect domestication processes.

## 2 Materials and Methods

### 2.1 Sampling Approach

Cacao specimens kept in the *ex‐situ* germplasm collection of the Brazilian Executive Commission for the Cacao Cultivation Plan (CEPLAC/Ministry of Agriculture in Itabuna, Bahia, Brazil) were sampled in August 2021. All sampled trees were cultivated and maintained at CEPLAC and were specifically chosen for their representation of the genetic‐geographic groups as defined in Motamayor et al. (2008). Each accession was labeled with unique identifiers linked to its geographic and genetic classification.

For the Guiana group, the exact accessions analyzed by Motamayor et al. (2008) were not available; however, we used accessions collected from the same river basin in French Guiana. Notably, extensive work has been conducted to characterize and conserve wild cacao populations in French Guiana, leading to the establishment of a core collection at the Centre de Coopération Internationale en Recherche Agronomique pour le Développement (CIRAD/Perennial Crops Biological Resource Centre, Sinnamary, French Guiana), which captures the full known diversity of the Guiana genetic group (Lachenaud et al. 2018). Table S1 provides the full description of sample origins and clone names, ensuring traceability with previous studies.

**FIGURE 1:**
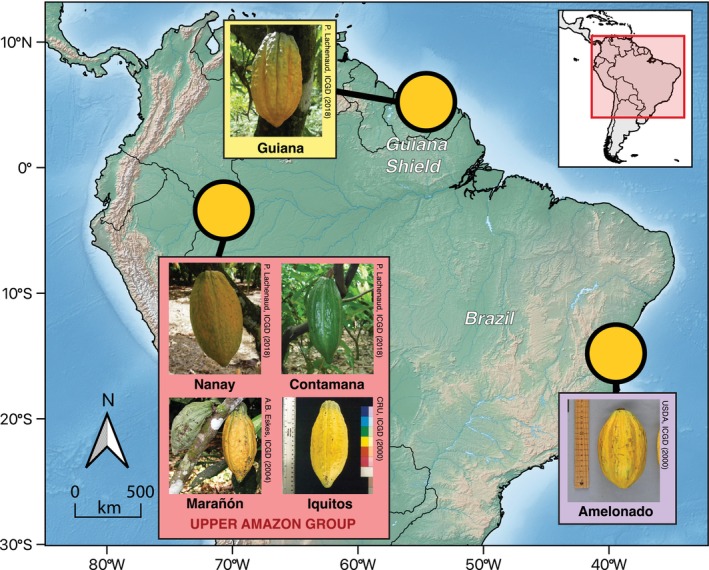
Approximate location of the populations of *Theobroma cacao* included in this study and representative images of cacao fruits from the Contamana (photo by E. Apshara), Guiana (photo by P. Lachenaud), Marañón (photo by P. Lachenaud), Amelonado (photo by USDA, ARS Tropical Agriculture Research Station) Nanay (photo by E. Apshara), and Iquitos (photo by CRU) genetic groups. Selected accessions are documented and accessible through the International Cocoa Germplasm Database (http://www.icgd.reading.ac.uk/).

For each accession, mature leaves were collected and preserved in silica gel. In total, 42 accessions were sampled, representing six populations across three major groups: Upper Amazonian, Guiana, and Amelonado. The Upper Amazonian group included 28 accessions from four populations as defined by Motamayor et al. (2008): Contamana (*n* = 8), Iquitos (*n* = 6), Marañón (*n* = 7), and Nanay (*n* = 7). The Guiana group comprised 8 accessions derived from a single river basin in French Guiana. The Amelonado group included 6 accessions that represent the introduced variety historically cultivated in Eastern Brazil. This sampling ensures broad coverage of the genetic‐geographic clusters relevant for our comparative analyses (Figure 1).

### 2.2 DNA Extraction, Sequencing and Variant Calling

Approximately 12–15 mg of ground, silica‐dried leaf tissue per sample was used for DNA extraction following the CTAB protocol of Doyle and Doyle (1987), with minor modifications. During the incubation step, 0.2% β‐mercaptoethanol was added to the extraction buffer. Samples were extracted three times with chloroform‐isoamyl alcohol (24:1), a modification of the original protocol intended to enhance the removal of contaminants, followed by ethanol precipitation using 100% and then 70% ethanol. DNA integrity was assessed by agarose gel electrophoresis, and concentrations were determined using a Qubit fluorometer (Thermo Fisher Scientific, Waltham, MA, USA). A total of 30 μL of DNA at 20 ng/μL per sample was submitted to Floragenex Inc. (Beaverton, OR, USA) for library preparation and sequencing.

RAD‐seq libraries were constructed using the restriction enzyme PstI and custom barcoded adapters designed for this enzyme. DNA was digested, sheared by sonication, ligated to adapters, and fragments of 300–500 bp were size‐selected and amplified. Sequencing was performed using 100 bp single‐end reads on an Illumina NovaSeq6000 platform, targeting a coverage of ~3–5× per sample for the RAD‐seq reads, consistent with shallow sequencing strategies for population‐level studies. RAD‐seq was selected for this study as a cost‐effective and scalable method to recover genome‐wide variants in a non‐model species such as *T. cacao* . Although reduced‐representation approaches like RAD‐seq can introduce biases (e.g., allele dropout, variable coverage, and limited resolution for linkage‐based analyses) (Andrews et al. 2016), they remain suitable for our goal of assessing broad‐scale population structure and genomic diversity patterns across multiple accessions.

Downstream analyses were conducted using R (R Core Team 2022) and Python environments. Raw sequence read files were demultiplexed and quality‐filtered using the “Stacks 2” pipeline (Rochette et al. 2019), specifically through the “process_radtags” function implemented via the “stackr” v. 2.2.0 in R (Gosselin et al. 2016). Reads were cleaned by removing low‐quality bases, ambiguous barcodes, and unique reads, and then normalized to 10 million reads per sample using the “FastqSampler” function in the “ShortRead” v. 4.2 package (Morgan et al. 2009). Read quality control was assessed using “FastQC” v. 0.1.2 and “QuasR” v. 1.38.0 (Gaidatzis et al. 2015). Sequences were aligned to the reference genome of *T. cacao* (Criollo cultivar B97‐61/B2; Argout et al. 2017) using “ShortRead” package. Variant calling was performed with “bcftools” v. 1.16 (Li et al. 2009), and filtering was applied using “vcftools” v. 0.1.16 (Danecek et al. 2011). Only biallelic SNPs were retained, and loci were filtered to include only those with a genotype call rate above 95%, a base quality score greater than 30, a minimum read depth of 5, and a minor allele count of at least 3. Individuals with high proportions of missing genotypes were excluded prior to downstream analyses to ensure data quality.

### 2.3 Population Genomic Analyses

To characterize the genetic diversity and structure of cacao populations, we performed multiple population genomic analyses using the SNP datasets derived from RAD‐sequencing. Nucleotide diversity (*π*), a measure of the average genetic variation within populations, was calculated for each group using “bcftools” v. 1.16 (Li et al. 2009). Windowed nucleotide diversity scores were computed at 10 kbp intervals across the genome. Tajima's *D* was calculated to assess deviation from neutral evolutionary expectation, identifying genomic regions potentially influenced by selection or demographic history. Both windowed nucleotide diversity and Tajima's *D* metrics were computed using “vcftools” v. 0.1.16 (Danecek et al. 2011) for each population and were compared statistically across groups using the Kruskal–Wallis test (at a significance level of 0.01).

We also evaluated homozygosity, heterozygosity and inbreeding coefficient to further explore genetic diversity and population structure. Observed heterozygosity (Ho), expected heterozygosity (He), and homozygosity levels per tree were estimated using “vcftools” directly from the genotype frequencies for each individual in the dataset, and the inbreeding coefficient (*F*) was derived to assess deviation between the observed and expected heterozygosity. These metrics provided additional insights into the level of genetic variation and potential signs of inbreeding within and between groups. Statistical comparisons (at a significance level of 0.01) were conducted to evaluate differences in these metrics.

To investigate broader patterns of population differentiation, we calculated pairwise genetic differentiation (*F*_ST_) using “vcftools”. *F*_ST_ estimates the proportion of genetic variance within populations relative to the overall variance. Windowed *F*_ST_ scores were computed at 10 kbp intervals and visualized to highlight regions of high differentiation across the genome. Statistical tests, including the pairwise Wilcoxon rank‐sum test, were applied to compare *F*_ST_ values across groups and evaluate the significance of observed differentiation.

Population structure was further assessed using principal component analysis (PCA) to identify clustering patterns among groups. PCA was performed using the “adegenet” package v. 2.1.10 in R (Jombart 2008; R Core Team 2022), which reduces the dimensionality of the genomic data while preserving genetic variance. The first two principal components were plotted to visualize genetic relationships among individuals from the Upper Amazon, Guiana, and Amelonado groups.

Finally, an ancestry group analysis was conducted in STRUCTURE v. 2.3.4 (Pritchard et al. 2000) to infer ancestry ratios and to detect potential admixture among populations. STRUCTURE runs were conducted across a range of potential ancestral clusters (*K*), varying from 1 to 8, with three replicates for each *K*. Each run included 20,000 Markov Chain Monte Carlo (MCMC) repetitions, following a burn‐in of 2000. Data visualization and determination of the optimal number of ancestral clusters (*K*) were conducted using “pophelper” v. 2.3.1 (Francis 2017) in R. Non‐parametric tests (Kruskal–Wallis and Wilcoxon rank‐sum) were used to assess statistical differences among groups, which do not require normality assumptions.

## 3 Results

The sequences included a total of 944,958 variant sites across the 42 accessions analyzed in the three groups. Genetic diversity of cacao populations was summarized using nucleotide diversity (*π*), Tajima's *D*, heterozygosity, homoztgosity, and inbreeding coefficients (Table 1). Significant differences were observed for nucleotide diversity (*p* < 0.01) and Tajima's *D* (*p* < 0.001) among the three groups. The Guiana group exhibited the highest nucleotide diversity (*π* = 1.775 ± 1.874 × 10^−5^, mean ± standard deviation), followed by the Amelonado group (*π* = 1.645 ± 1.668 × 10^−5^) and Upper Amazonian populations (*π* = 1.146 ± 0.978 × 10^−5^). Tajima's *D* values were highest in Upper Amazonian populations (2.881 ± 0.644), followed by Amelonado (2.403 ± 0.711) and Guiana (1.788 ± 0.714). Appendix S1 (Supporting Information—S1) contains the full output files for these two analyses. While there is some variation in nucleotide diversity and heterozygosity among Upper Amazonian populations, they are generally similar and reflect their shared evolutionary background and designation as wild in previous classifications.

**TABLE 1:** Summary of nucleotide diversity (*π*), Tajima's *D*, observed heterozygosity (Ho), expected heterozygosity (He), inbreeding coefficient (F), and homozygosity per tree for the three cacao groups: Upper Amazonian populations (Contamana, Iquitos, Marañón (*n* = 7), and Nanay), Guiana, and Amelonado.

Variable	Groups (*N* = 42)			*H*
	Upper Amazonian (*n* = 28)	Guiana (*n* = 8)	Amelonado (*n* = 6)	
Nucleotide diversity (×10^−5^)	1.146 ± 0.978	1.775 ± 1.874	1.645 ± 1.668	163.25*
Tajima's *D*	2.881 ± 0.644	1.788 ± 0.714	2.403 ± 0.711	3542.6**
Observed heterozygosity	0.395 ± 0.159	0.532 ± 0.251	0.480 ± 0.340	1.694^ns^
Expected heterozygosity	0.365 ± 0.051	0.489 ± 0.183	0.473 ± 0.221	2.028^ns^
Inbreeding coefficient	−0.066 ± 0.383	−0.063 ± 0.234	0.100 ± 0.366	1.597^ns^
Homozygosity per tree	0.776 ± 0.126	0.789 ± 0.159	0.773 ± 0.201	0.322^ns^

*Note:* H = Kruskal–Wallis' chi‐squared rank sum test scores. Statistical significance of pairwise comparisons is indicated by asterisks: *p < 0.01, **p < 0.001, ns = not significant (*p* ≥ 0.01).

Observed heterozygosity (Ho) was highest in Guiana (0.532 ± 0.251), followed by Amelonado (0.480 ± 0.340) and Upper Amazonian populations (0.395 ± 0.159). Expected heterozygosity (He) followed a similar trend, with Guiana (0.489 ± 0.183) and Amelonado (0.473 ± 0.221) displaying similar values, whereas Upper Amazonian populations exhibited slightly lower levels (0.365 ± 0.051). Inbreeding coefficients (*F*) were negative for both Guiana (−0.063 ± 0.234) and Upper Amazonian populations (−0.066 ± 0.383), indicating an excess of heterozygotes relative to Hardy–Weinberg expectations. In contrast, Amelonado showed a slightly positive F value (0.100 ± 0.366). Kruskal‐Wallis tests revealed no statistically significant differences among groups for Ho (*p* = 0.249), He (*p* = 0.363), or *F* (*p* = 0.151) (Table 1). Additionally, comparisons between observed and expected heterozygosity within each group indicated no significant deviation (Wilcoxon signed‐rank test: *p* = 1 for Amelonado and Guiana, *p* = 0.350 for Upper Amazonian populations). Appendix S2 (Supporting Information—S1) shows the full output files for these two analyses.

To further evaluate whether Amelonado accessions exhibit increased homozygosity consistent with self‐compatibility, we quantified individual‐level homozygosity across groups. Mean homozygosity per accession was 0.776 ± 0.126 for Upper Amazonian, 0.789 ± 0.159 for Guiana, and 0.773 ± 0.201 for Amelonado individuals. Kruskal–Wallis tests revealed no significant difference across groups (*H* = 2.265, *p* = 0.322; Table 1), suggesting broadly similar genome‐wide homozygosity levels and no strong evidence for elevated selfing in Amelonado under our sampling.

**FIGURE 2:**
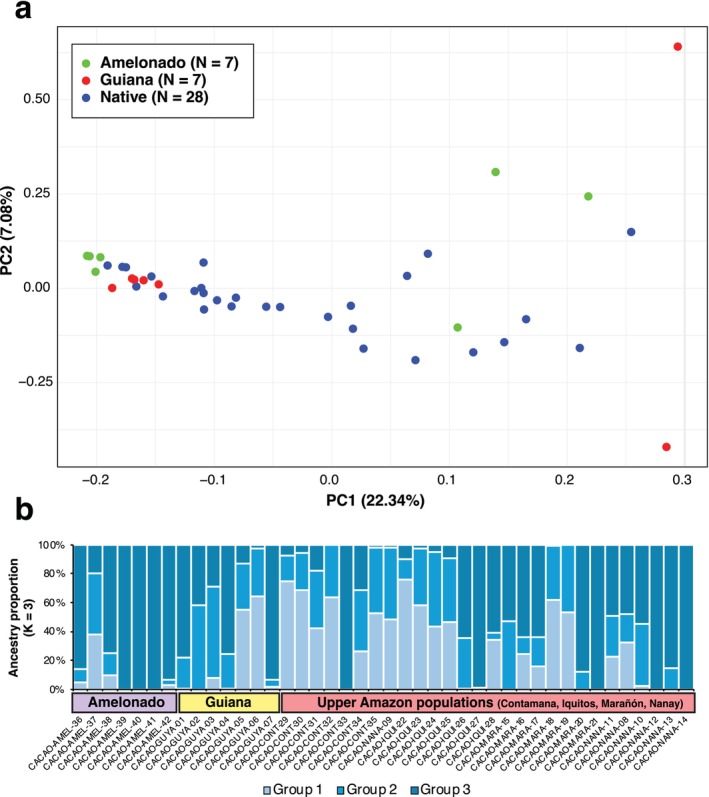
Population structure and genetic ancestry of cacao groups. (a) Principal Component Analysis (PCA) of genetic variation among Upper Amazonian populations, Guiana, and Amelonado groups. Each point represents an individual, with colors indicating group membership: Upper Amazonian (blue), Guiana (green), and Amelonado (orange). The PCA highlights the broad genetic diversity within Upper Amazonian populations, tight clustering of Guiana samples, and the distinct separation of Amelonado, consistent with its domesticated status. (b) STRUCTURE analysis showing ancestry proportions for the three groups at *K* = 3 (optimal ancestry group recovered). Each vertical bar represents an individual, divided into color‐coded segments corresponding to inferred genetic clusters. The Upper Amazonian group shows admixture from multiple clusters, Guiana exhibit predominantly wild‐like ancestry with minimal admixture, and Amelonado is dominated by a single distinct cluster, reflecting its domestication history and limited genetic diversity.

Principal Component Analysis (PCA) of all individuals revealed clustering patterns that correspond to the three groups (Figure 2a). The Amelonado group generally clustered together, while Upper Amazonian individuals exhibited broad dispersion along both principal components. The Guiana group displayed a tighter cluster with partial overlap with some Upper Amazonian individuals. A few individuals positioned between groups or outside cluster cores suggest intermediate patterns or outliers within the dataset. Appendix S3 in the Supporting Information—S1 contains the output files associated with these analyses.

Pairwise *F*_ST_ comparisons (Table 2) revealed low differentiation between Upper Amazonian populations and the Guiana group (*F*_ST_ = 0.026 ± 0.209), suggesting a close genetic relationship. Differentiation between Upper Amazonian and Amelonado populations was lower (*F*_ST_ = 0.001 ± 0.183), consistent with the domestication history of the latter. The highest differentiation was observed between Guiana and Amelonado populations (mean *F*_ST_ = 0.043 ± 0.269). Wilcoxon rank‐sum tests confirmed statistical differences between Guiana and Amelonado populations (*p* < 0.001) and between Upper Amazonian and Amelonado populations (*p* < 0.001), while no significant differentiation was observed between the Upper Amazonian and the Guiana group (*p* = 0.200). The results of these analyses are available in Appendix S4, included as Supporting Information—S1.

**TABLE 2:** Mean *F*_ST_ values (± standard deviation, S.D) between Upper Amazonian, Guiana, and Amelonado groups, with *p*‐values for the Wilcoxon rank‐sum test for statistical differences for each comparison. Low *F*_ST_ values between Upper Amazonian and Guiana groups suggest minimal differentiation, while higher values for Amelonado reflect distinct genetic divergence due to domestication.

Comparison	Mean *F*_ST_ ± SD	*p*
Upper Amazonian vs. Guiana	0.026 ± 0.209	0.200
Upper Amazonian vs. Amelonado	< 0.001 ± 0.183	< 0.001
Guiana vs. Amelonado	0.043 ± 0.269	< 0.001

Finally, STRUCTURE analyses were performed across *K* = 1–8, and *K* = 3 was identified as the optimal number of ancestry clusters based on likelihood score comparisons (Appendix S5). The Amelonado group was largely composed of a single ancestry component (Cluster 3 in Figure 2b), while Guiana individuals mostly exhibited a shared component with Upper Amazonian individuals (Cluster 2), but with some signs of admixture. Upper Amazonian populations displayed the most mixed ancestry, with contributions from all three clusters and considerable individual variation. These patterns are generally consistent with the PCA (Figure 2a) but also highlight underlying admixture not fully captured in principal component space.

## 4 Discussion

### 4.1 Genetic Diversity and Population Structure

Our study offers new insights into the genetic diversity and structure of cacao populations across the Amazon basin, highlighting the interplay between natural processes and human influence in shaping their genetic composition. While our sampling does not encompass the full diversity of cacao across its entire range, our findings suggest that the Upper Amazonian populations represent true wild populations. These groups are characterized by high nucleotide diversity, heterozygosity, and balancing selection (based on Tajima's *D* values), aligning with their recognition as the primary center of cacao's genetic diversity (Cornejo et al. 2018; Fouet et al. 2022; Silva et al. 2011). These attributes also underscore the Upper Amazon basin's role in preserving cacao's genetic diversity, serving as a reservoir for future breeding and conservation efforts. However, we acknowledge that our RAD‐seq dataset does not capture genomic features such as structural variants, long‐range linkage disequilibrium, or polygenic signatures detectable in full‐genome or transcriptome‐based studies, as the study of Hämälä et al. (2021, 2020).

Although the Upper Amazonian group comprises multiple subpopulations (e.g., Contamana, Marañón, Iquitos, and Nanay), grouping them together follows established knowledge in cacao population genetics (Motamayor et al. 2008; Cornejo et al. 2018). These populations have been shown to share similar evolutionary histories and high genetic diversity, and their collective treatment as a wild ancestral group is supported by prior genomic studies (Cornejo et al. 2018; Fouet et al. 2022; Motamayor et al. 2008). However, the strong influence of human‐mediated dispersal—through introductions, cultivation, and trade—may in some cases obscure or outpace natural selection signals, complicating the interpretation of population genomic patterns such as Tajima's *D* and heterozygosity (Pickrell and Reich 2014).

Secondly, our results corroborate that the Amelonado group exhibits signatures consistent with domestication, including elevated Tajima's *D* and a positive inbreeding coefficient, which reflect demographic contraction and reduced outcrossing. While it shows intermediate values for nucleotide diversity and heterozygosity—higher than Upper Amazonian but lower than Guiana populations—these patterns still align with its known history of introduction, self‐compatibility, and propagation from a narrow genetic base (Bartley 2005; Santos et al. 2015; Wood and Lass 2008). Historical records suggest that Amelonado cacao was first introduced to Bahia in the mid‐18th century from seedlings originating from Pará, likely from material previously cultivated by settlers and Jesuit missionaries, who may have facilitated early cultivation in the Lower Amazon before its dispersal to Bahia (Bartley 2005; Santos et al. 2015; Vello and Garcia 1971).

The Amelonado cacaos are known to be mostly self‐compatible, a trait that may have been favored during domestication to ensure increased pod set in cultivated stands (Cope 1976). Cope (1976) hypothesized that genotypes occurring near the putative center of diversity in the Upper Amazon would predominantly be self‐incompatible, whereas self‐compatible trees would become more frequent in populations further from this region. The prevalence of self‐compatibility in Amelonado may thus reflect early selection pressures favoring plants capable of setting fruit with minimal pollination. This characteristic, combined with artificial selection for other traits (e.g., more and larger seeds), likely contributed to the genetic homogeneity observed in Amelonado cacao and its widespread cultivation beyond the Amazon basin.

The Guiana group, however, reveals a unique and intriguing genetic profile. Contrary to an alternative hypothesis suggesting their origins as human‐mediated introductions (Colli‐Silva et al. 2024), our results indicate that they are more consistent with isolated wild populations. The intermediate nucleotide diversity, clustering patterns in PCA and STRUCTURE analyses, and minimal genetic differentiation from Upper Amazonian populations all point to an early origin for these populations. Importantly, these findings reinforce earlier conclusions by Lachenaud et al. (2004) and Lachenaud and Zhang (2008), which had already suggested that the Guiana might harbor wild cacao populations. The STRUCTURE results further support this interpretation, revealing shared genetic ancestry between the Guiana and Upper Amazonian populations, in contrast to the distinct ancestry profiles of the Amelonado group.

Statistical analyses reinforce these conclusions. Pairwise *F*_ST_ comparisons showed significant differentiation between Amelonado and both Upper Amazonian and Guiana groups (*p* < 0.001), consistent with the introduced nature of Amelonado. In contrast, the lack of significant differentiation between the Upper Amazonian and the Guiana group (*p* = 0.200) suggests closer genetic affinity between these groups. Similarly, Tajima's *D* values for the Guiana reflect neutral or weakly positive selection, aligning more closely with the expected for wild populations rather than with recently introduced groups. These patterns collectively suggest that the Guiana group may be geographically isolated wild populations, retaining much of the genetic structure of Upper Amazonian populations while remaining less influenced by human‐mediated selection processes. In population genetics, positive Tajima's *D* values—such as those observed in our Upper Amazonian and Amelonado populations—may indicate an excess of intermediate‐frequency alleles, which can arise from processes like balancing selection, historical population contractions, or bottlenecks (Nielsen 2005; Simonsen et al. 1995; Tajima 1989). In the context of cacao, these elevated values are likely a reflection of complex demographic histories shaped by both natural evolutionary forces and even pre‐Columbian stages of domestication.

Although PCA and STRUCTURE analysis both revealed major group‐level trends, we acknowledge that neither shows complete genetic isolation between groups. Several individuals appeared in intermediate positions in the PCA, particularly between Guiana and Upper Amazonian populations, highlighting the continuous nature of cacao genetic variation. Similarly, the STRUCTURE bar plots show variable ancestry contributions across individuals, especially among Upper Amazonian populations, which reflect admixture and shared ancestral components. These findings align with the complex demographic and evolutionary history of cacao and suggest that group boundaries, while useful for summarizing patterns, do not represent strictly discrete populations.

Our group‐level findings also complement recent investigations into polygenic adaptation and genome architecture in cacao. Hämälä et al. (2020) demonstrated that local adaptation can leave subtle signatures across functionally related gene modules rather than isolated loci, while their follow‐up study (Hämälä et al. 2021) revealed that structural variants may constrain or facilitate adaptation depending on their effect on gene regulation and recombination. Although our RAD‐seq approach is not designed to detect such variants, the admixture and partial clustering patterns observed in our PCA and STRUCTURE analyses could reflect similar underlying processes.

### 4.2 Rethinking the “Created Centers” of Cacao

The term “created center” refers to regions where cacao diversity has arisen not through natural evolutionary processes, but through human‐mediated mixing, selection, or dispersion of genetic material—resulting in distinct, secondary centers of diversity (see, e.g., Harlan 1971). The Guiana group represents a potential genetic resource of cacao that remains underexplored in breeding programs. Previous studies suggested that these populations could be relics of ancient wild cacao rather than recent human introductions (Lachenaud et al. 2004; Lachenaud and Zhang 2008) and, as discussed earlier, our findings reinforce such status, indicating that these populations may have been preserved in situ due to natural geographic barriers. Additionally, Thevenin et al. (2012) and Lachenaud et al. (2015, 2018) proposed that Guiana populations form a distinct genetic cluster, separate from Upper Amazonian populations, but without clear evidence of domestication traits.

Our results also emphasize how domestication shaped cacao's genetic structure across regions. Historical and genetic studies indicate that domestication primarily influenced distinct groups such as Criollo, Amelonado, and Trinitario, each associated with unique histories and genetic profiles (Motamayor et al. 2002, 2003). Criollo, cultivated by pre‐Columbian civilizations in Mesoamerica, has low genetic diversity levels (Cornejo et al. 2018), while Amelonado, originating from the Lower Amazon, shows reduced variation relative to wild Upper Amazonian populations. These genetic bottlenecks were likely driven by events such as the introduction of Amelonado to Bahia in Brazil, and its subsequent spread to West Africa (Bartley 2005).

Beyond the Guianas, the genetic composition of cacao populations in the Lower Amazon also provides some insights into cacao's evolutionary and domestication history. Sereno et al. (2006) demonstrated that Lower Amazonian populations exhibit greater genetic variation than previously assumed, suggesting they may include remnants of early domesticated lineages or have experienced gene flow from Upper Amazonian groups. This challenges the idea that the Lower Amazon was solely a secondary center of diversity derived from early human introductions. Furthermore, Cope (1976) proposed that self‐incompatible genotypes predominate in the Upper Amazon, whereas self‐compatible trees become more frequent in regions farther from this area, reinforcing the idea that cacao's historical geography was a mosaic of genetic exchanges, local adaptations, and human‐mediated selection.

Amelonado provides a clear example of how human‐mediated selection shaped cacao's genetic diversity. Introduced to Eastern Brazil in the mid‐18th century (Bartley 2005; Vello and Garcia 1971), this variety exhibits reduced genetic diversity and higher inbreeding due to its propagation from a limited self‐compatible genetic base (Santos et al. 2015). Self‐compatibility, a trait likely favored during domestication to ensure fruit set with minimal pollination (Cope 1976), would have contributed to Amelonado's widespread cultivation but also to its genetic homogeneity. Molecular analyses confirm that Bahian cacaos, including the SIAL and SIC clones (early selected Amelonado lineages maintained in germplasm collections as representatives of Bahian cacao diversity), represent a genetically narrow subset of Amelonado cacao (Santos et al. 2015). This genetic restriction, coupled with intense artificial selection for productivity and disease resistance, facilitated its expansion across Brazil's Atlantic coast but also increased susceptibility to pathogens such as witches' broom in Bahia, mirroring patterns seen in West African Amelonado populations (Lopes et al. 2022; Santos et al. 2015).

Although Amelonado is expected to exhibit elevated homozygosity due to its self‐compatible nature and domestication bottlenecks, our genome‐wide estimates of per‐individual homozygosity did not differ significantly from those of Upper Amazonian or Guiana individuals. These findings suggest that, despite reduced diversity and inbreeding, Amelonado populations in our dataset may not exhibit notably higher levels of selfing at the genome scale (Table 1), or that such patterns may be confounded by other demographic or sampling factors. Given its historical significance and continued role in breeding programs, further investigation into the genetic structure of Amelonado populations would be important for conservation and improvement efforts.

The Trinitario cultivar, a hybrid between Criollo and Forastero, provides another example of the “created center” concept, where human intervention combined distinct genetic lineages. “Forastero,” a term historically used in opposition to Criollo, actually refers to a broad and genetically diverse set of populations native to the Amazon basin. Emerging in Trinidad following a disease outbreak that devastated Criollo plantations in the 18th century, the Trinitario cacao combined the disease resistance and yield of Amelonado with the quality traits of Criollo (Cheesman 1944; Pittier 1935). However, plastid DNA analyses (Yang et al. 2013) suggest Trinitario's origin was more complex than previously thought, involving multiple independent introductions and subsequent hybridization between Upper and Lower Amazonian Forasteros in addition to Criollo. These findings reinforce that cacao's domestication and dispersal were shaped by repeated human‐mediated events rather than a single introduction event followed by local adaptation. Understanding these complex domestication processes provides critical context for interpreting the genetic structure of the Guiana populations, which may similarly reflect a mix of dispersal, hybridization, and selection.

In summary, expanded sampling across the Amazon Basin is essential to better define the boundaries between wild and introduced cacao populations. While our findings support the wild status of the Guiana group, other regions, such as the Lower Amazon basin, remain largely unexplored and may harbor populations with ambiguous histories. Integrating genetic, archaeological, and ecological data will be key to distinguishing truly wild populations from those shaped by human influence. Understanding these populations is crucial for conservation and breeding efforts, particularly as cacao faces growing environmental challenges. Future studies should prioritize comparative genomic analyses with related *Theobroma* species, such as *cupuaçu* (Colli‐Silva et al. 2023) and others (Bossa‐Castro et al. 2024), alongside multidisciplinary approaches to reconstruct cacao's evolutionary history. Protecting wild populations, particularly those potentially wild, is vital for ensuring the resilience, adaptability, and long‐term sustainability of cultivated cacao, securing its economic and cultural significance worldwide.

## Author Contributions

**Matheus Colli‐Silva:** conceptualization (lead), formal analysis (lead), investigation (lead), methodology (lead), writing – original draft (lead). **James Edward Richardson:** supervision (lead), validation (lead), writing – review and editing (equal). **José Rubens Pirani:** funding acquisition (lead), supervision (lead), writing – review and editing (equal). **Antonio Figueira:** supervision (equal), validation (lead), writing – review and editing (equal).

## Conflicts of Interest

The authors declare no conflicts of interest.

## Supporting information


**Appendix S1:** Output files for nucleotide diversity (*π*) and Tajima’s *D* analyses, providing detailed metrics for population‐level genetic variation across all samples.
**Appendix S2:** Output files for expected heterozygosity (He), observed heterozygosity (Ho), and fixation index (*F*), summarizing genetic diversity and inbreeding coefficients across the sampled populations.
**Appendix S3:** Principal Component Analysis (PCA) outputs, including loadings and coordinates for all individuals sampled, highlighting population structure and genetic clustering patterns.
**Appendix S4:** Pairwise FST output files, detailing genetic differentiation metrics between sampled populations, reflecting gene flow and isolation patterns.
**Appendix S5:** STRUCTURE analysis outputs, detailing ancestry proportions and admixture patterns for K values ranging from 1 to 8. Here are included the output files for each run and replicate, a summary of likelihood values for all tested conditions, and a graph illustrating the variation in likelihoods as *K* ranges from 1 to 8. These results provide insights into population genetic structure and the extent of admixture among groups.
**Table S1:** Traceable matchings between the sample names used in this study and the clone designations reported by Motamayor et al. (2008), providing a clear reference for sample identity and origin. * = Not sampled in Motamayor et al. (2008), but sample coming from the same collection and the same region (see Bartley 2005).

## Data Availability

Raw FASTQ sequencing files are available at the NCBI Sequence Read Archive (SRA) (accession number PRJNA948463) and as variant call files (VCFs) at the European Variation Archive (EVA) under accession number PRJEB61107. Supporting Information—S1 related to this manuscript is also available at https://figshare.com/s/03047aeccb8bd51370c7.
