# Supplementary figures and images for: Wild or Introduced? Investigating the Genetic Landscape of Cacao Populations in South America
Source: Ecol Evol. 2025 Jul 22;15(7):e71746. doi: 10.1002/ece3.71746 (PMC12283127; doi:10.1002/ece3.71746)

Expected Heterozygosity

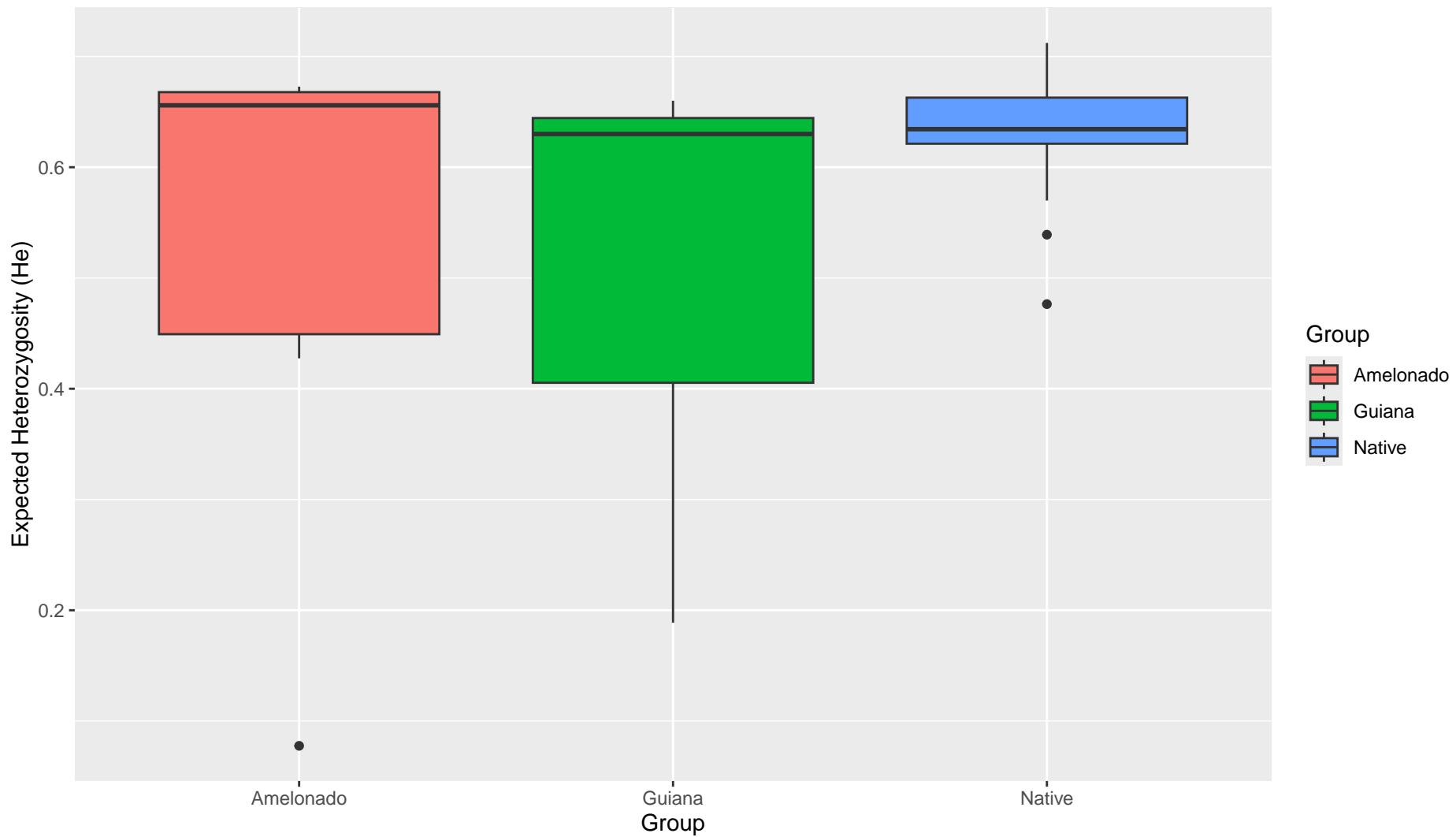

Supplement: Supplementary file 1 — Appendix S1: Output files for nucleotide diversity (π) and Tajima’s D analyses, providing detailed metrics for population‐level genetic variation across all samples. Appendix S2: Output files for expected heterozygosity (He), observed heterozygosity (Ho), and fixation index (F), summarizing genetic diversity and inbreeding coefficients across the sampled populations. Appendix S3: Principal Component Analysis (PCA) outputs, including loadings and coordinates for all individuals sampled, highlighting population structure and genetic clustering patterns. Appendix S4: Pairwise FST output files, detailing genetic differentiation metrics between sampled populations, reflecting gene flow and isolation patterns. Appendix S5: STRUCTURE analysis outputs, detailing ancestry proportions and admixture patterns for K values ranging from 1 to 8. Here are included the output files for each run and replicate, a summary of likelihood values for all tested conditions, and a graph illustrating the variation in likelihoods as K ranges from 1 to 8. These results provide insights into population genetic structure and the extent of admixture among groups. Table S1: Traceable matchings between the sample names used in this study and the clone designations reported by Motamayor et al. (2008), providing a clear reference for sample identity and origin. * = Not sampled in Motamayor et al. (2008), but sample coming from the same collection and the same region (see Bartley 2005). [file ECE3-15-e71746-s001.zip › Supplementary Materials/Appendix_S2/Expected Heterozigosity.pdf]

Observed Heterozygosity

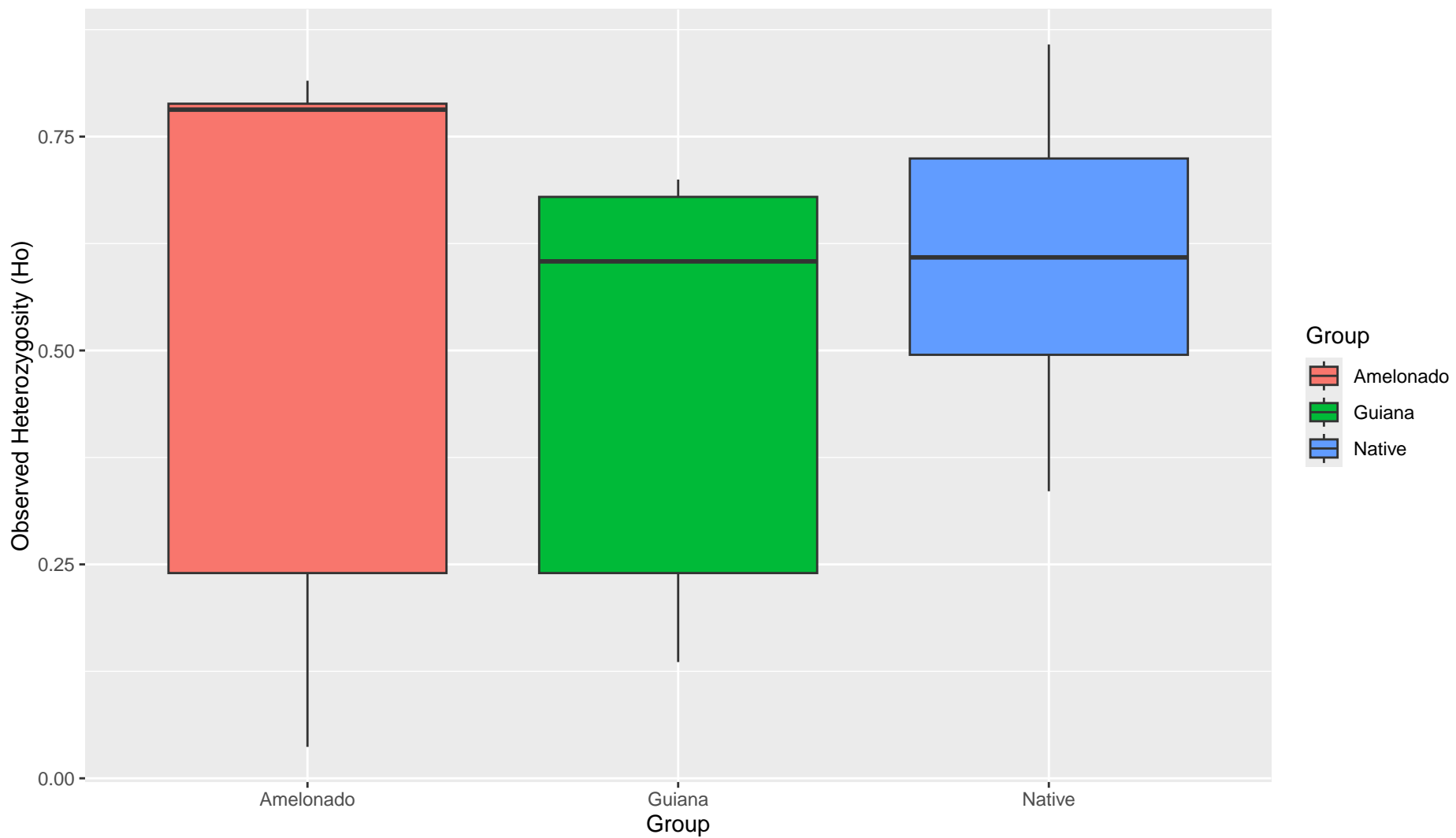

Supplement: Supplementary file 1 — Appendix S1: Output files for nucleotide diversity (π) and Tajima’s D analyses, providing detailed metrics for population‐level genetic variation across all samples. Appendix S2: Output files for expected heterozygosity (He), observed heterozygosity (Ho), and fixation index (F), summarizing genetic diversity and inbreeding coefficients across the sampled populations. Appendix S3: Principal Component Analysis (PCA) outputs, including loadings and coordinates for all individuals sampled, highlighting population structure and genetic clustering patterns. Appendix S4: Pairwise FST output files, detailing genetic differentiation metrics between sampled populations, reflecting gene flow and isolation patterns. Appendix S5: STRUCTURE analysis outputs, detailing ancestry proportions and admixture patterns for K values ranging from 1 to 8. Here are included the output files for each run and replicate, a summary of likelihood values for all tested conditions, and a graph illustrating the variation in likelihoods as K ranges from 1 to 8. These results provide insights into population genetic structure and the extent of admixture among groups. Table S1: Traceable matchings between the sample names used in this study and the clone designations reported by Motamayor et al. (2008), providing a clear reference for sample identity and origin. * = Not sampled in Motamayor et al. (2008), but sample coming from the same collection and the same region (see Bartley 2005). [file ECE3-15-e71746-s001.zip › Supplementary Materials/Appendix_S2/Observed Heterozigosity.pdf]

Inbreeding Coefficient (F)

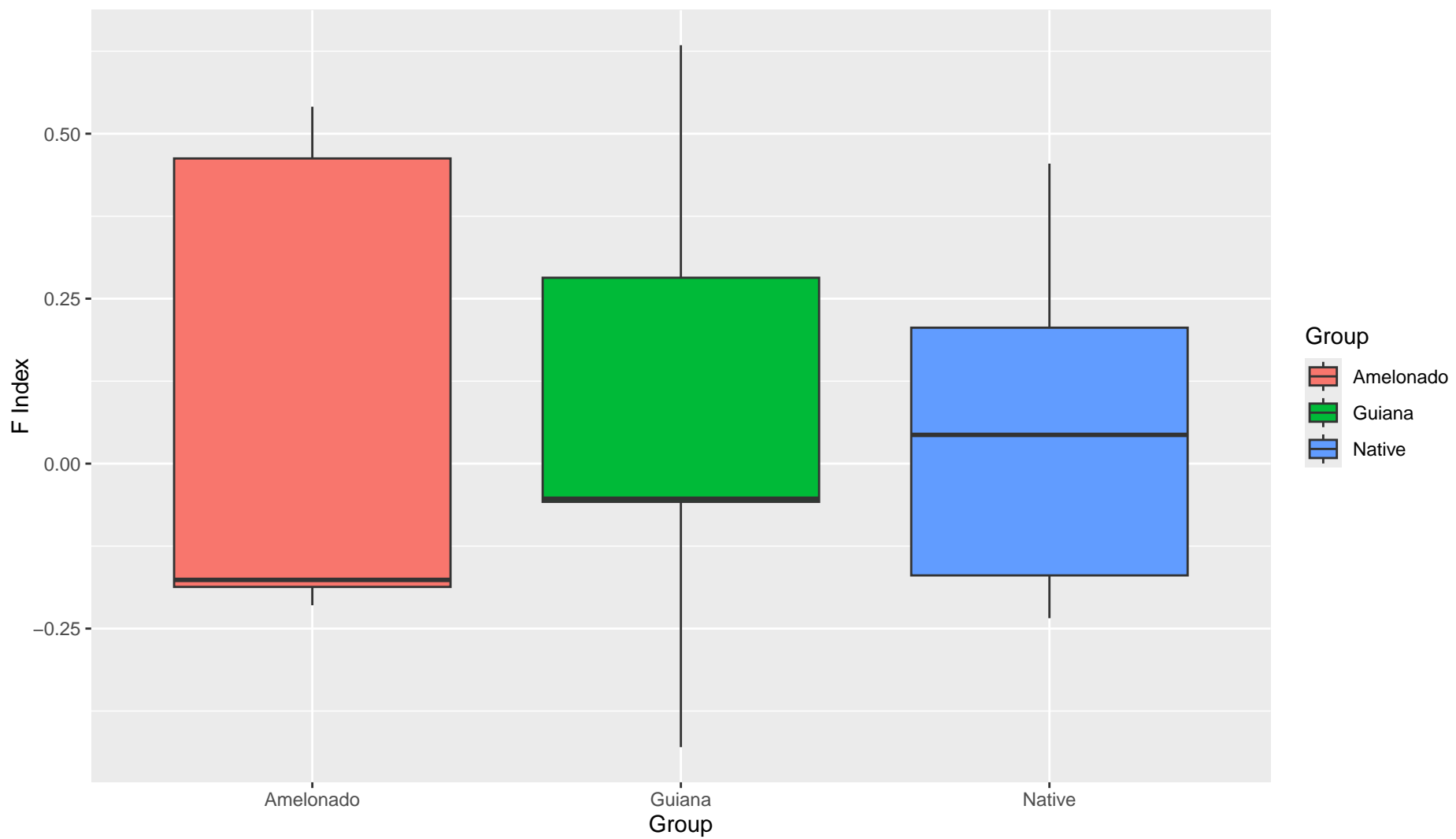

Supplement: Supplementary file 1 — Appendix S1: Output files for nucleotide diversity (π) and Tajima’s D analyses, providing detailed metrics for population‐level genetic variation across all samples. Appendix S2: Output files for expected heterozygosity (He), observed heterozygosity (Ho), and fixation index (F), summarizing genetic diversity and inbreeding coefficients across the sampled populations. Appendix S3: Principal Component Analysis (PCA) outputs, including loadings and coordinates for all individuals sampled, highlighting population structure and genetic clustering patterns. Appendix S4: Pairwise FST output files, detailing genetic differentiation metrics between sampled populations, reflecting gene flow and isolation patterns. Appendix S5: STRUCTURE analysis outputs, detailing ancestry proportions and admixture patterns for K values ranging from 1 to 8. Here are included the output files for each run and replicate, a summary of likelihood values for all tested conditions, and a graph illustrating the variation in likelihoods as K ranges from 1 to 8. These results provide insights into population genetic structure and the extent of admixture among groups. Table S1: Traceable matchings between the sample names used in this study and the clone designations reported by Motamayor et al. (2008), providing a clear reference for sample identity and origin. * = Not sampled in Motamayor et al. (2008), but sample coming from the same collection and the same region (see Bartley 2005). [file ECE3-15-e71746-s001.zip › Supplementary Materials/Appendix_S2/Imbreeding Coefficient F.pdf]

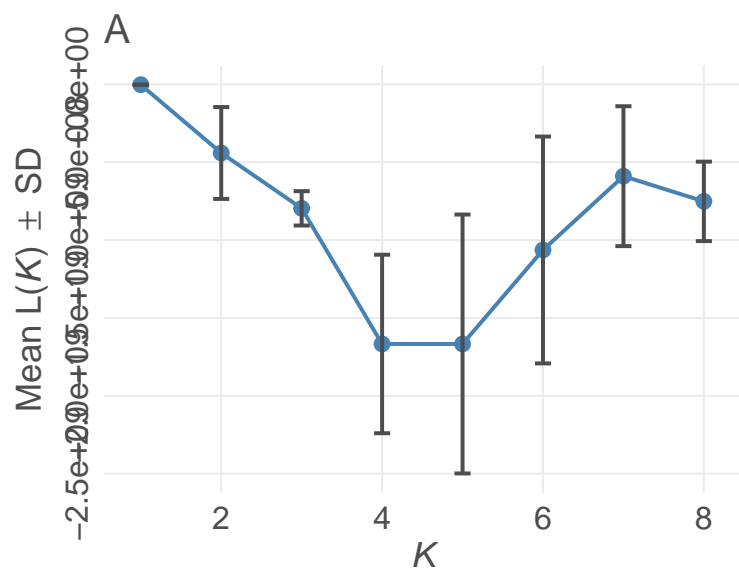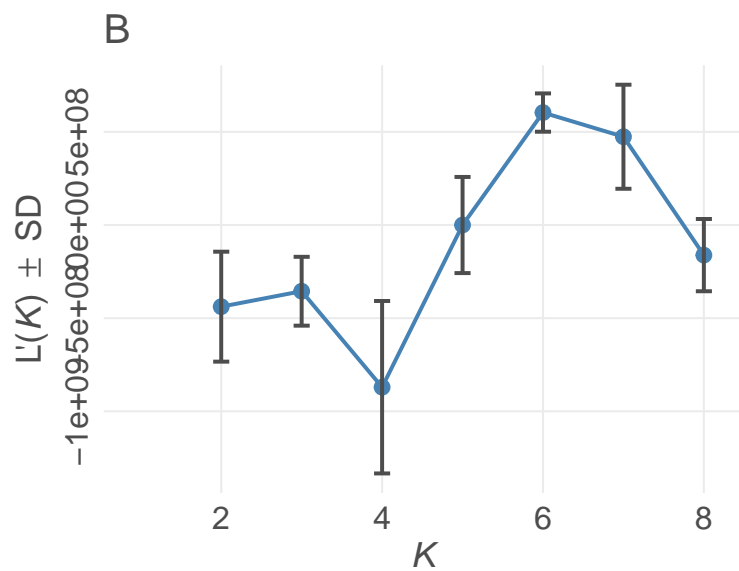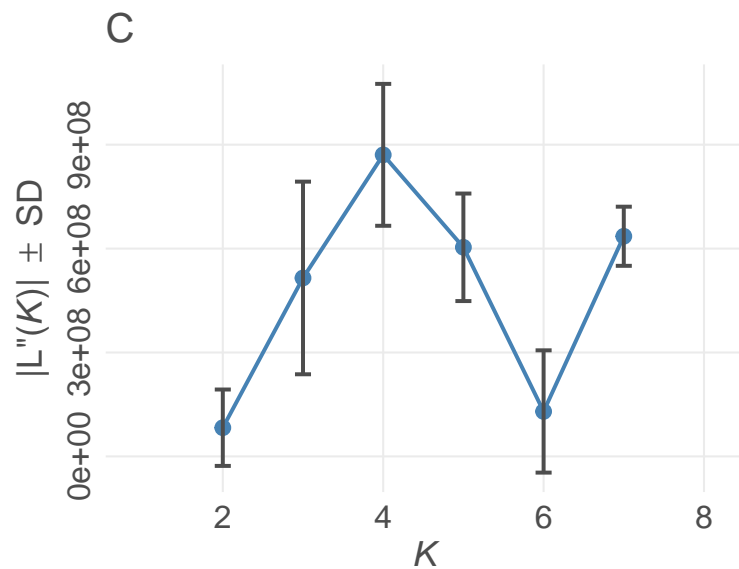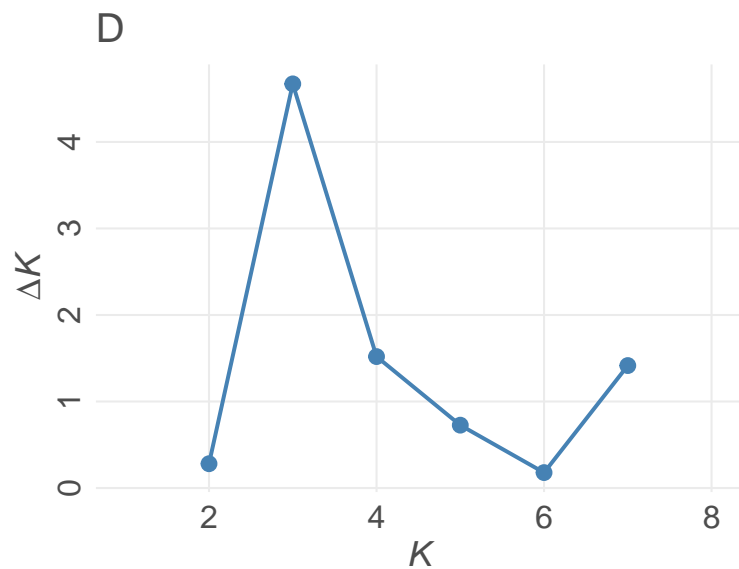

Supplement: Supplementary file 1 — Appendix S1: Output files for nucleotide diversity (π) and Tajima’s D analyses, providing detailed metrics for population‐level genetic variation across all samples. Appendix S2: Output files for expected heterozygosity (He), observed heterozygosity (Ho), and fixation index (F), summarizing genetic diversity and inbreeding coefficients across the sampled populations. Appendix S3: Principal Component Analysis (PCA) outputs, including loadings and coordinates for all individuals sampled, highlighting population structure and genetic clustering patterns. Appendix S4: Pairwise FST output files, detailing genetic differentiation metrics between sampled populations, reflecting gene flow and isolation patterns. Appendix S5: STRUCTURE analysis outputs, detailing ancestry proportions and admixture patterns for K values ranging from 1 to 8. Here are included the output files for each run and replicate, a summary of likelihood values for all tested conditions, and a graph illustrating the variation in likelihoods as K ranges from 1 to 8. These results provide insights into population genetic structure and the extent of admixture among groups. Table S1: Traceable matchings between the sample names used in this study and the clone designations reported by Motamayor et al. (2008), providing a clear reference for sample identity and origin. * = Not sampled in Motamayor et al. (2008), but sample coming from the same collection and the same region (see Bartley 2005). [file ECE3-15-e71746-s001.zip › Supplementary Materials/Appendix_S5/Variation_K.pdf]

# FST by Comparison

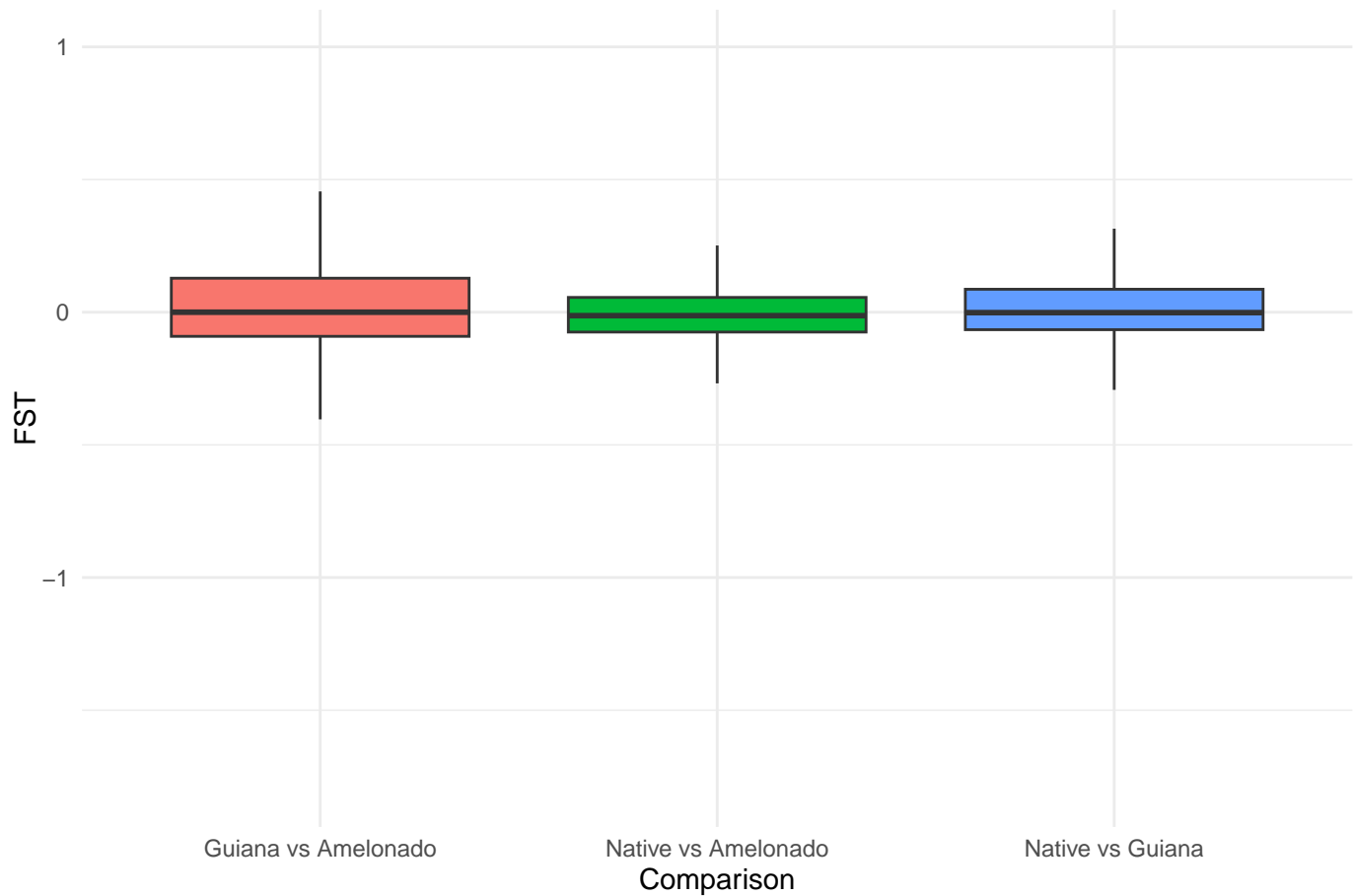

Supplement: Supplementary file 1 — Appendix S1: Output files for nucleotide diversity (π) and Tajima’s D analyses, providing detailed metrics for population‐level genetic variation across all samples. Appendix S2: Output files for expected heterozygosity (He), observed heterozygosity (Ho), and fixation index (F), summarizing genetic diversity and inbreeding coefficients across the sampled populations. Appendix S3: Principal Component Analysis (PCA) outputs, including loadings and coordinates for all individuals sampled, highlighting population structure and genetic clustering patterns. Appendix S4: Pairwise FST output files, detailing genetic differentiation metrics between sampled populations, reflecting gene flow and isolation patterns. Appendix S5: STRUCTURE analysis outputs, detailing ancestry proportions and admixture patterns for K values ranging from 1 to 8. Here are included the output files for each run and replicate, a summary of likelihood values for all tested conditions, and a graph illustrating the variation in likelihoods as K ranges from 1 to 8. These results provide insights into population genetic structure and the extent of admixture among groups. Table S1: Traceable matchings between the sample names used in this study and the clone designations reported by Motamayor et al. (2008), providing a clear reference for sample identity and origin. * = Not sampled in Motamayor et al. (2008), but sample coming from the same collection and the same region (see Bartley 2005). [file ECE3-15-e71746-s001.zip › Supplementary Materials/Appendix_S4/fst_native_guiana_is_ns_rest_less_0001.pdf]

PCA of Combined Groups

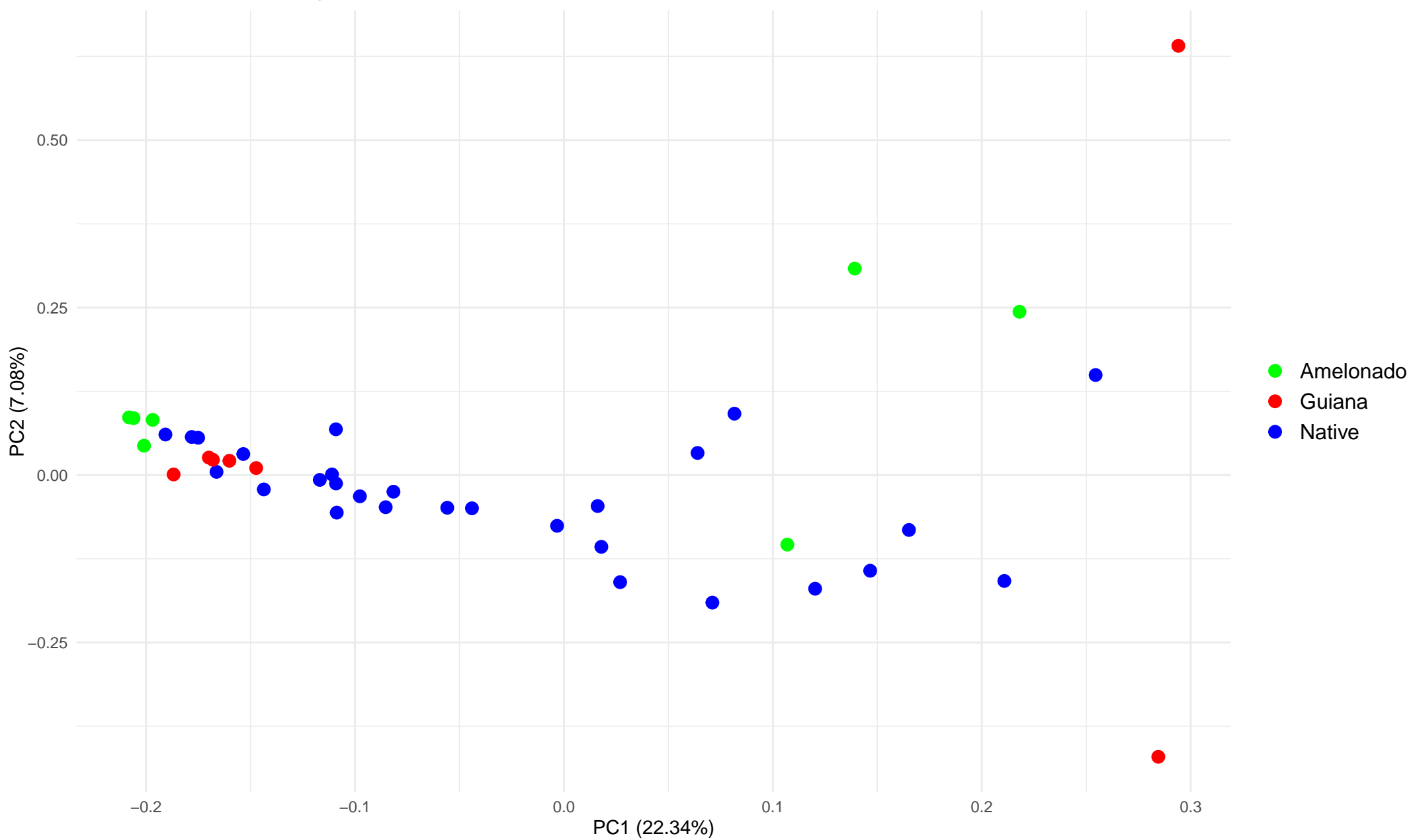

Supplement: Supplementary file 1 — Appendix S1: Output files for nucleotide diversity (π) and Tajima’s D analyses, providing detailed metrics for population‐level genetic variation across all samples. Appendix S2: Output files for expected heterozygosity (He), observed heterozygosity (Ho), and fixation index (F), summarizing genetic diversity and inbreeding coefficients across the sampled populations. Appendix S3: Principal Component Analysis (PCA) outputs, including loadings and coordinates for all individuals sampled, highlighting population structure and genetic clustering patterns. Appendix S4: Pairwise FST output files, detailing genetic differentiation metrics between sampled populations, reflecting gene flow and isolation patterns. Appendix S5: STRUCTURE analysis outputs, detailing ancestry proportions and admixture patterns for K values ranging from 1 to 8. Here are included the output files for each run and replicate, a summary of likelihood values for all tested conditions, and a graph illustrating the variation in likelihoods as K ranges from 1 to 8. These results provide insights into population genetic structure and the extent of admixture among groups. Table S1: Traceable matchings between the sample names used in this study and the clone designations reported by Motamayor et al. (2008), providing a clear reference for sample identity and origin. * = Not sampled in Motamayor et al. (2008), but sample coming from the same collection and the same region (see Bartley 2005). [file ECE3-15-e71746-s001.zip › Supplementary Materials/Appendix_S3/PCA groups.pdf]

Tajima's D Across the Genome

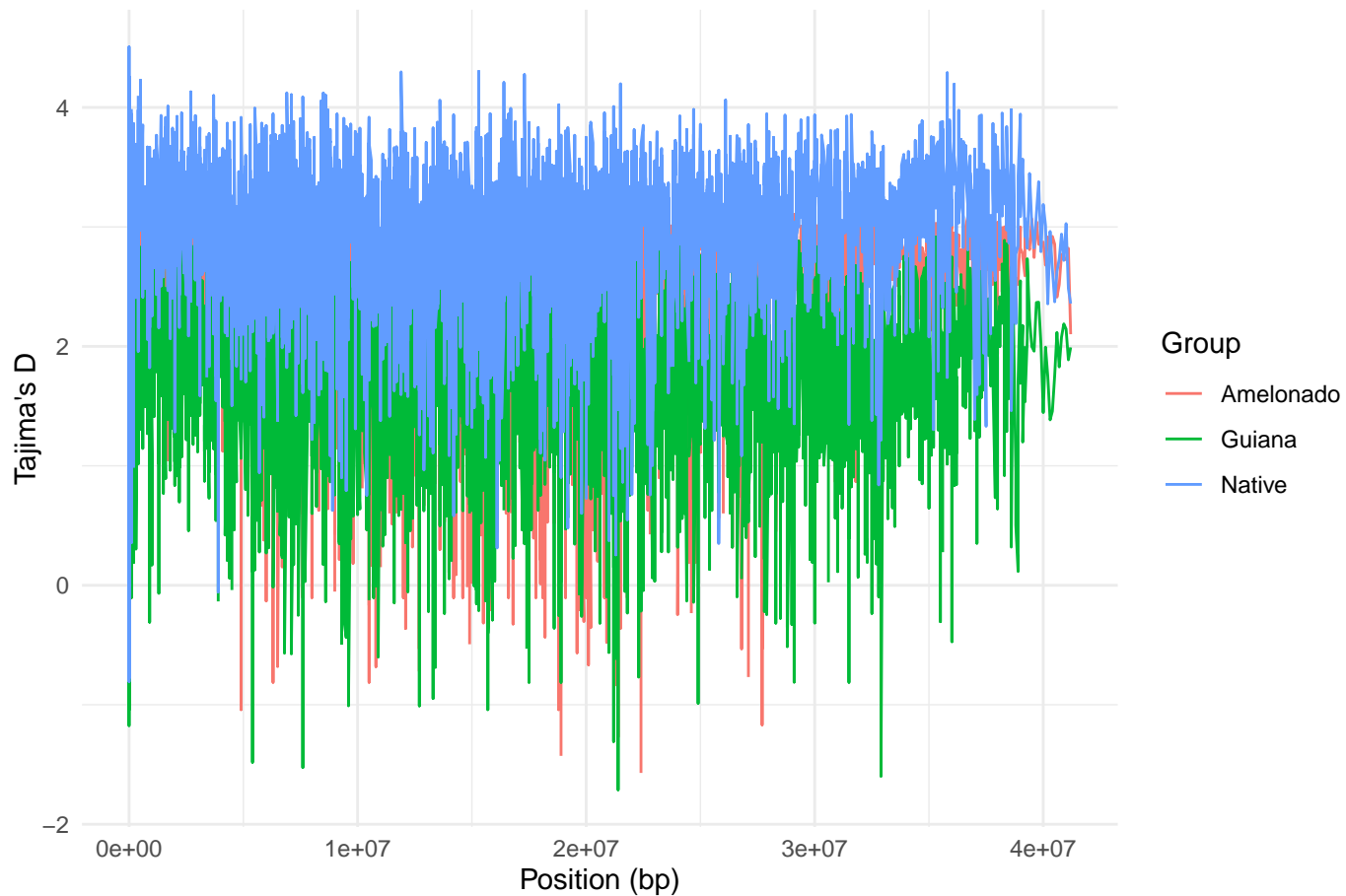

Supplement: Supplementary file 1 — Appendix S1: Output files for nucleotide diversity (π) and Tajima’s D analyses, providing detailed metrics for population‐level genetic variation across all samples. Appendix S2: Output files for expected heterozygosity (He), observed heterozygosity (Ho), and fixation index (F), summarizing genetic diversity and inbreeding coefficients across the sampled populations. Appendix S3: Principal Component Analysis (PCA) outputs, including loadings and coordinates for all individuals sampled, highlighting population structure and genetic clustering patterns. Appendix S4: Pairwise FST output files, detailing genetic differentiation metrics between sampled populations, reflecting gene flow and isolation patterns. Appendix S5: STRUCTURE analysis outputs, detailing ancestry proportions and admixture patterns for K values ranging from 1 to 8. Here are included the output files for each run and replicate, a summary of likelihood values for all tested conditions, and a graph illustrating the variation in likelihoods as K ranges from 1 to 8. These results provide insights into population genetic structure and the extent of admixture among groups. Table S1: Traceable matchings between the sample names used in this study and the clone designations reported by Motamayor et al. (2008), providing a clear reference for sample identity and origin. * = Not sampled in Motamayor et al. (2008), but sample coming from the same collection and the same region (see Bartley 2005). [file ECE3-15-e71746-s001.zip › Supplementary Materials/Appendix_S1/Tajima_D/TajimaD_whole_genome.pdf]

Tajima's D by Group

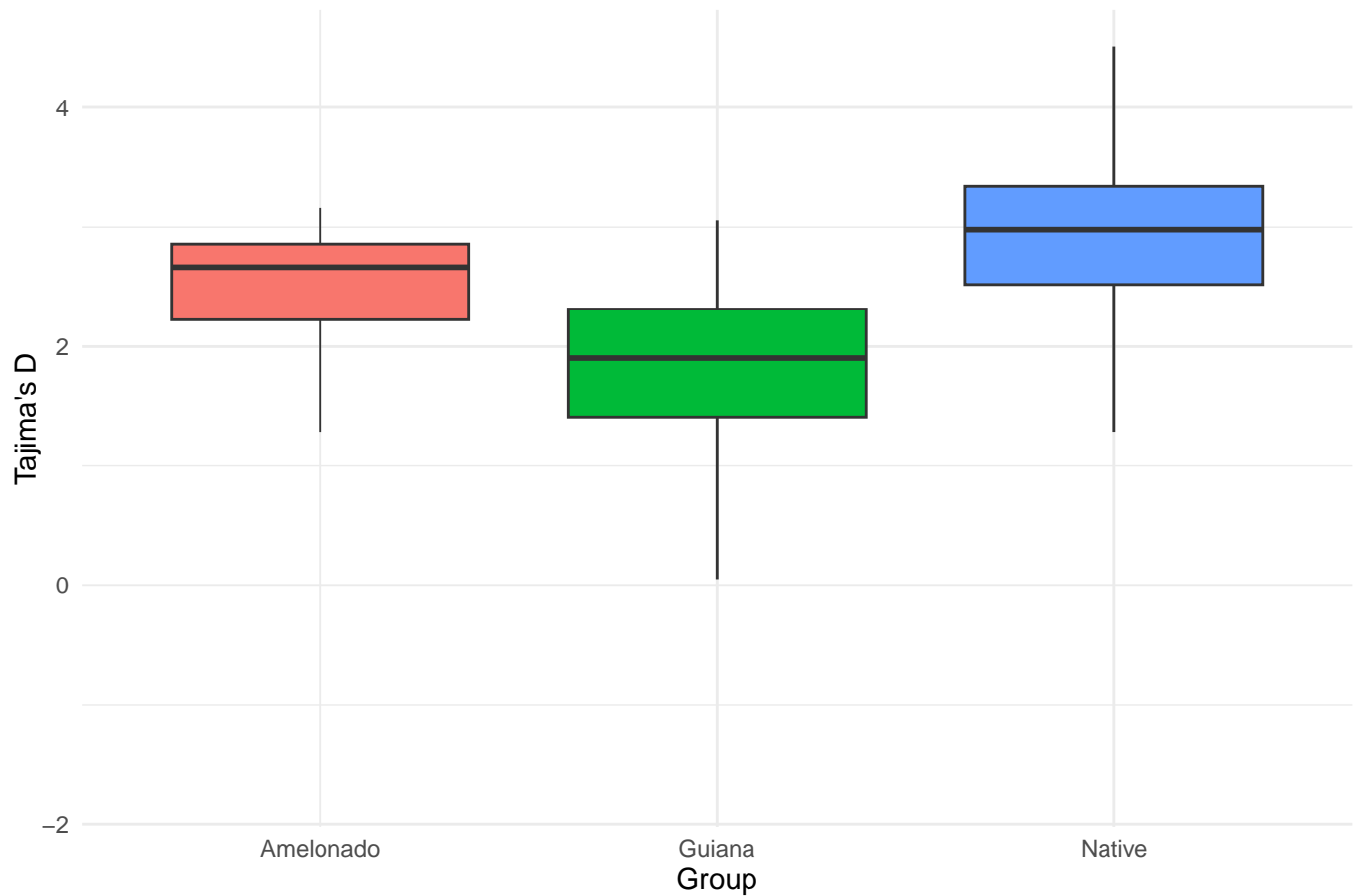

Supplement: Supplementary file 1 — Appendix S1: Output files for nucleotide diversity (π) and Tajima’s D analyses, providing detailed metrics for population‐level genetic variation across all samples. Appendix S2: Output files for expected heterozygosity (He), observed heterozygosity (Ho), and fixation index (F), summarizing genetic diversity and inbreeding coefficients across the sampled populations. Appendix S3: Principal Component Analysis (PCA) outputs, including loadings and coordinates for all individuals sampled, highlighting population structure and genetic clustering patterns. Appendix S4: Pairwise FST output files, detailing genetic differentiation metrics between sampled populations, reflecting gene flow and isolation patterns. Appendix S5: STRUCTURE analysis outputs, detailing ancestry proportions and admixture patterns for K values ranging from 1 to 8. Here are included the output files for each run and replicate, a summary of likelihood values for all tested conditions, and a graph illustrating the variation in likelihoods as K ranges from 1 to 8. These results provide insights into population genetic structure and the extent of admixture among groups. Table S1: Traceable matchings between the sample names used in this study and the clone designations reported by Motamayor et al. (2008), providing a clear reference for sample identity and origin. * = Not sampled in Motamayor et al. (2008), but sample coming from the same collection and the same region (see Bartley 2005). [file ECE3-15-e71746-s001.zip › Supplementary Materials/Appendix_S1/Tajima_D/boxplots_allp_less_0001.pdf]
